# The Influence of Age on the Likelihood of Catheter-Free Fistula Use in Hemodialysis Patients

**DOI:** 10.1177/2054358119861943

**Published:** 2019-07-30

**Authors:** Farah Ladak, Pietro Ravani, Matthew J. Oliver, Fareed Kamar, Alix Clarke, Swapnil Hiremath, Jennifer MacRae, Peter Blake, Louise M. Moist, Amit X. Garg, Ngan Lam, Chance Dumaine, Robert R. Quinn

**Affiliations:** 1 Cumming School of Medicine, University of Calgary, AB, Canada; 2 Department of Community Health Sciences, University of Calgary, AB, Canada; 3 Division of Nephrology, Department of Medicine, University of Toronto, ON, Canada; 4 Division of Nephrology, Department of Medicine and Kidney Research Centre, Ottawa Hospital Research Institute, University of Ottawa, ON, Canada; 5 Kidney Clinical Research Unit, London Health Sciences Centre, ON, Canada; 6 Division of Nephrology, Department of Medicine, Western University, London, ON, Canada; 7 Institute for Clinical Evaluative Sciences, Toronto, ON, Canada; 8 Division of Nephrology, Department of Medicine, University of Alberta, Edmonton, Canada

**Keywords:** chronic kidney disease, hemodialysis, arteriovenous fistula, central venous catheter

## Abstract

**Background:**

Clinical practice guidelines recommend arteriovenous fistulas as the preferred form of vascular access for hemodialysis. However, some studies have suggested that older age is associated with poorer fistula outcomes.

**Objective:**

We assessed the impact of age on the outcomes of fistula creation and access-related procedures.

**Design:**

This was a prospective cohort study using data collected as part of the Dialysis Measurement Analysis and Reporting (DMAR) system.

**Setting:**

Participating Canadian dialysis programs, including Southern Alberta Renal Program, Manitoba Renal Program, Sunnybrook Health Sciences Centre (Toronto, Ontario), London Health Sciences Centre (London, Ontario), and The Ottawa Hospital (Ottawa, Ontario).

**Patients:**

Incident hemodialysis patients aged 18 years and older who started dialysis between January 1, 2004, and May 31, 2012.

**Measurements:**

The primary outcome was the proportion of all first fistula attempts that resulted in catheter-free fistula use, defined as independent use of a fistula for hemodialysis (ie, no catheter in place). Secondary outcomes included the time to catheter-free fistula use among patients with a fistula creation attempt, total number of days of catheter-free fistula use, and the proportion of a patient’s hemodialysis career spent with an independently functioning fistula (ie, catheter-free fistula use).

**Methods:**

We compared patient characteristics by age group, using *t* tests or Wilcoxon rank sum tests, and chi-square or Fisher exact tests, as appropriate. Logistic and fractional logistic regression were used to estimate the odds of achieving catheter-free fistula use by age group and the proportion of dialysis time spent catheter-free, respectively.

**Results:**

A total of 1091 patients met our inclusion criteria (567 age ≥ 65; 524 age < 65). Only 57% of first fistula attempts resulted in catheter-free fistula use irrespective of age (adjusted odds ratio [OR]_≥65vs<65_: 1.01; *P* = .93). The median time from hemodialysis start to catheter-free use of the first fistula did not differ by age when grouped into fistulas attempted pre- and post-dialysis initiation. The adjusted rates of access-related procedures were comparable (incidence rate ratio [IRR]_≥65vs<65:_ 0.95; *P* = .32). The median percentage of follow-up time spent catheter-free was similar and low in patients who attempted fistulas (<65 years: 19% vs ≥65 years: 21%; *P* = .85).

**Limitations:**

The relatively short follow-up time may have underestimated the benefits of fistula creation and the observational study design precludes inferences about causality.

**Conclusions:**

In our study, older patients who underwent a fistula attempt were just as likely as younger patients to achieve catheter-free fistula use, within a similar time frame, and while requiring a similar number of access procedures. However, the minority of dialysis time was spent catheter-free.

## Abrégé

**Contexte:**

Les lignes directrices cliniques recommandent de privilégier la fistule artérioveineuse comme accès vasculaire pour l’hémodialyse. Certaines études suggèrent toutefois que les résultats seraient moins bons chez les patients âgés.

**Objectif:**

Nous avons examiné l’effet de l’âge du patient sur l’issue de la création d’une fistule et sur les procédures liées à l’accès.

**Type d’étude:**

Il s’agit d’une étude de cohorte prospective utilisant les données colligées par le système DMAR (*Dialysis Measurement Analysis and Reporting*).

**Cadre:**

Les programmes de dialyse canadiens participants, soit le *Southern Alberta Renal Program*, le *Manitoba Renal Program*, le Sunnybrook Health Sciences Centre (Toronto, Ontario), le London Health Sciences Centre (London, Ontario), et l’hôpital d’Ottawa (Ottawa, Ontario).

**Sujets:**

Les patients adultes incidents ayant amorcé une hémodialyse entre le 1^er^ janvier 2004 et le 31 mai 2012.

**Mesures:**

La principale mesure était la proportion de premières fistules créées ayant mené à une utilisation sans cathéter, soit à un usage indépendant pour l’hémodialyse. Les mesures secondaires incluaient le temps écoulé jusqu’à l’utilisation d’une fistule sans cathéter pour les patients ayant subi une première tentative, le nombre total de jours d’utilisation d’une fistule sans cathéter, et la proportion du temps de dialyse passé avec une fistule indépendante fonctionnelle (sans cathéter).

**Méthodologie:**

Nous avons comparé les caractéristiques des patients par groupe d’âge à l’aide de tests t ou de tests de somme des rangs de Wilcoxon, et de tests chi-deux ou de tests exacts de probabilité de Fisher, selon le cas. Une régression logistique et une régression logistique fractionnée ont été employées pour estimer respectivement, selon le groupe d’âge, les chances d’utiliser une fistule sans cathéter et la proportion du temps de dialyse passé sans cathéter.

**Résultats:**

Au total, 1 091 patients satisfaisaient nos critères d’inclusion (n=567 [≥65 ans]; n=524 [<65 ans]). Seulement 57 % des premières tentatives de création d’une fistule ont mené à une utilisation sans cathéter, indépendamment de l’âge (rapport de cote corrigé [RC]_≥65contre<65:_ 1,01; p=0,93). Le temps médian jusqu’à l’utilisation sans cathéter de la première fistule créée n’a pas varié en fonction de l’âge lorsque les patients ont été groupés selon que la fistule avait été créée avant ou après l’amorce de la dialyse. Les taux corrigés de procédures liées à l’accès vasculaire étaient similaires (rapport des taux d’incidence [RTI]_≥65contre<65_ 0,95; p=0,32); tout comme le pourcentage médian de temps de dialyse passé sans cathéter qui s’est avéré faible pour tous les patients (19 % [<65 ans] contre 21 % [≥65 ans]; p=0,85).

**Limites:**

La période de suivi relativement courte pourrait avoir sous-estimé les avantages de créer une fistule, et la nature observationnelle de l’étude ne permet pas de tirer de conclusions sur la causalité.

**Conclusion:**

Selon notre étude, les patients âgés avaient autant de chance que les plus jeunes d’utiliser la fistule sans cathéter, et ce, dans un délai semblable et avec sensiblement le même nombre de procédures liées à l’accès vasculaire. Néanmoins, la proportion du temps de dialyse passé sans cathéter était faible.

## What was known before

Half of all fistulas created will experience failure within the first year of creation. Fistula-related complications, including the failure to mature to adequately support dialysis, can lead to invasive interventions that reduce patient quality of life, and consume significant radiological and surgical resources. Some studies have suggested that this risk is higher among adults over the age of 65 years and this patient population makes up a large proportion of those starting dialysis in developed countries. There is increasing interest in taking a more patient-centered approach to the selection of vascular access. To inform decision making, there was a need to better quantify the potential risks and benefits of different vascular access strategies and to determine whether or not they are influenced by age.

## What this adds

In our study, 57% of patients who attempted fistula creation went on to catheter-free use of their fistulas. Older patients who underwent a fistula attempt were just as likely as younger patients to achieve catheter-free fistula use in a similar time frame. The duration of fistula use and the proportion of patients’ dialysis careers spent catheter-free were similar, regardless of age, as were adjusted procedure rates. Only 19% to 21% of the time spent on hemodialysis was catheter-free following a first fistula creation. This increased to 36% to 40% if multiple attempts at fistula creation were allowed and to 73% to 75% if analyses were restricted to a selected cohort that had at least 3 years of follow-up.

## Introduction

Hemodialysis is the most common form of renal replacement therapy for individuals with end-stage renal disease (ESRD) and requires reliable access to the blood stream. Central venous catheters (“catheters”), arteriovenous fistulas (“fistulas”), and arteriovenous grafts (“grafts”) are the 3 main options for vascular access.^1^ Clinical practice guidelines strongly recommend fistulas because they are associated with a lower risk of morbidity and mortality, and lower costs to establish and maintain patency in observational studies.^2-4^

Unfortunately, nearly half of all fistulas created will experience failure within the first year of creation, and some studies have suggested that the risk is higher among adults over the age of 65 years.^5,6^ Fistula-related complications, including the failure to mature to adequately support dialysis, can lead to invasive interventions that reduce patient quality of life, consume significant radiological and surgical resources, and ultimately result in the use of a catheter.^7-9^ Older patients make up a large proportion of those starting dialysis in developed countries and there is increasing interest in taking a patient-centered approach to the selection of vascular access.^10-12^ To inform decision making, it is important to better quantify the potential risks and benefits of different vascular access strategies and to determine whether or not they are influenced by age.

We conducted a large, multicenter study to determine the impact of age on the outcomes of fistula creation. Specifically, we examined whether older patients (65 years of age and older) had a lower proportion of first fistula attempts that lead to catheter-free fistula use, longer time required to achieve catheter-free fistula use, or smaller proportion of time on hemodialysis that was catheter-free compared with younger patients. We also assessed the association between age and rates of access-related procedures.

## Materials and Methods

### Design and Setting

We used data from 5 Canadian dialysis programs (Southern Alberta Renal Program, Manitoba Renal Program, Sunnybrook Health Sciences Centre [Toronto, Ontario], London Health Sciences Centre [London, Ontario], and The Ottawa Hospital [Ottawa, Ontario]) that participated in the Dialysis Measurement Analysis and Reporting (DMAR) system. DMAR was a centrally hosted, web-based data collection system that prospectively collected detailed data on incident hemodialysis patients for quality improvement purposes. All data entry personnel were trained front-line staff who coded information using a standardized coding schema. Two investigators double reviewed all data and any queries were resolved in consultation with the end-user prior to data export and analysis. Data elements collected include baseline demographic, comorbidity, and laboratory information as well as any changes in dialysis modality, hospitalizations, transplants, losses to follow-up, transfers out of the program, and deaths. All vascular access procedures, before and after the initiation of dialysis, were captured along with the location, date, description, and indication for each procedure. The study protocol was approved by the research ethics boards at each participating institution.

### Study Population

**Figure 1.**
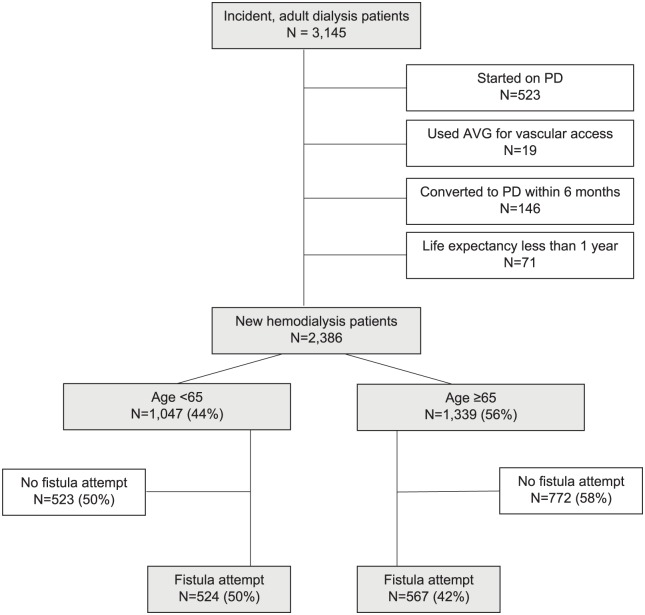
Cohort creation. Note. PD = peritoneal dialysis; AVG = arteriovenous grafts.

Incident hemodialysis patients aged 18 years and older who started dialysis between January 1, 2004, and May 31, 2012, were identified. Patients were included if they had a diagnosis of ESRD according to a nephrologist, received a single outpatient dialysis treatment, or received dialysis for a period of 28 days or more after an episode of acute kidney injury. Patients initiated either hemodialysis or continuous renal replacement therapy (CRRT) during the study period, and underwent at least 1 fistula attempt before or after the initiation of dialysis therapy. They were excluded if they used an arteriovenous graft at any time (grafts were uncommon in our cohort), used peritoneal dialysis (PD) in the first 6 months of renal replacement therapy, or had a life expectancy of less than 1 year at the time of dialysis initiation (metastatic cancer, or other terminal illness where death was imminent) (Figure 1). Patients were followed until the earliest of recovery of kidney function, receipt of a kidney transplant, transfer to PD, loss to follow-up, transfer out of the program, death, or the end of the study period (August 31, 2012).

### Age

The main exposure of interest was age, which we dichotomized into “< 65 years” and “≥65 years” groups. A cutoff of 65 years was selected as it has commonly been used in prior studies^13-15^ and because approximately half of the incident patients in the participating programs were over the age of 65.

### Outcomes

The primary outcome was the proportion of all first fistula attempts that resulted in catheter-free fistula use, defined as independent use of a fistula for hemodialysis (ie, no catheter in place). In situations where a patient had a catheter in place when they started dialysis or had a fistula attempt, catheter-free fistula use began when the catheter was removed and the fistula was used as the sole access for hemodialysis. This outcome was chosen because it was the most relevant marker of successful fistula creation, as the expressed intent of fistula creation is to spare patients from the potential harms of catheters. For our primary analysis, we defined it as catheter-free use of the first fistula and follow-up was censored at the time of a second fistula attempt, if applicable. A secondary analysis looked at catheter-free use of any fistula, allowing for multiple attempts in the same patient.

Secondary outcomes included the time to catheter-free fistula use among patients with a fistula creation attempt, total number of days of catheter-free fistula use, and the proportion of a patient’s hemodialysis career spent with an independently functioning fistula (ie, catheter-free fistula use). Time to catheter-free use was calculated in 2 ways depending on whether the fistula was created before dialysis initiation (“pre-dialysis”) or after dialysis initiation (“post-dialysis”), with time at risk beginning from the start of dialysis or date of fistula creation, respectively. For proportion of follow-up time spent catheter-free, follow-up time began at dialysis start (with timing of fistula creation accounted for in the modeling). We also calculated the rates of access procedures during the follow-up period among all patients undergoing a fistula creation (starting from the date of their first procedure, ie, fistula creation or catheter insertion to begin dialysis). Access procedures were subcategorized as either catheter-related, fistula-related (excluding creations), or fistula creations. Catheter-related procedures included catheter insertions, removals, exchanges, as well as fibrin sheath disruptions, central vein angioplasties, and line-o-grams. Fistula-related procedures included collateral vein embolization and ligation, fistulograms, angioplasties, fistula revisions, occlusions, and removals, thrombectomies and thrombolysis procedures, angiograms, and arterioplasties. Fistula creations included any form of fistula creation (radiocephalic, brachiocephalic, brachiobasilic, etc).

### Statistical Analysis

We compared patient characteristics by age group, using *t* tests or Wilcoxon rank sum tests, and chi-square or Fisher exact tests, as appropriate. Crude medians were presented to summarize catheter-free use outcomes. We then used a logistic regression model to estimate the odds ratio (OR) for achieving catheter-free fistula use by age group, and a fractional logistic regression model for proportions of catheter-free use. Crude intervention rates, per person-year, were calculated for each group as the total number of procedures divided by total follow-up time. Following assessment for distributional assumptions and the presence of excess zeroes, we used a Poisson model, or in the case of overdispersion, a negative binomial model to estimate adjusted incidence rate ratios (IRR). All regression models were adjusted for sex, body mass index (BMI), diabetes mellitus, coronary artery disease, congestive heart failure, peripheral vascular disease, cerebrovascular disease, and whether the fistula was created pre- or post-dialysis initiation. In our sensitivity analyses, we repeated the primary analysis after restricting our cohort to those with at least 3 years of follow-up. Next, we restricted the cohort to those who achieved catheter-free use of their fistulas at some point during follow-up to provide an estimate for catheter-free time in those who had successful fistula attempts. Finally, we categorized age as <65, 65-75, and 75+ to see whether it would impact our results.

## Results

A total of 3145 adult patients started dialysis during the period of interest. Five hundred twenty-three started dialysis on PD; 19 used an arteriovenous graft for vascular access; 146 intended to do PD and converted within the first 6 months of therapy; and 71 had a life expectancy of less than 1 year. A total of 1091 patients met criteria for inclusion in the study and underwent an attempt at fistula creation (Figure 1). Baseline characteristics of the study participants are presented in Table 1, stratified by age. There was a higher prevalence of comorbid conditions in the older cohort, with the exception of diabetes mellitus, which was more common in younger patients. Older patients had a lower BMI, higher estimated glomerular filtration rate (eGFR) at the start of dialysis, and were more likely to have received at least 12 months of predialysis care. The median follow-up time did not significantly differ by age group, averaging 1.9 years (see Table 2). For predialysis fistula attempts, follow-up from dialysis start was 1.8 years and for postdialysis fistula attempts, follow-up from dialysis start was 2.0 years (1.6 years from date of creation). Older patients were more likely to attempt their first fistula prior to the start of dialysis (43% in those <65 vs 50% in those ≥65; *P* value = .01). Finally, a total of 9% of patients <65 years of age died during follow-up compared with 34% of those ≥65 years of age.

**Table 1.** Baseline Characteristics of Patients Who Had a Fistula Attempt.

	All patients N = 1091	Age < 65 n = 524	Age ≥ 65 n = 567	*P* value
Age, median (IQR)	65 (55-75)	55 (45-60)	75 (70-80)	<.01*
BMI, median (IQR)	27.3 (23.7-32.2)	28.5 (24.2-34.2)	26.5 (23.5-30.8)	<.01*
Male, n (%)	688 (63)	342 (65)	346 (61)	.15
Diabetes mellitus, n (%)	646 (59)	332 (63)	314 (55)	<.01*
Coronary artery disease, n (%)	348 (32)	114 (22)	234 (41)	<.01*
Congestive heart failure, n (%)	234 (21)	79 (15)	155 (27)	<.01*
Cerebrovascular disease, n (%)	144 (13)	44 (8)	100 (18)	<.01*
Peripheral vascular disease, n (%)	162 (15)	57 (11)	105 (19)	<.01*
Cancer, n (%)	166 (15)	39 (7)	127 (22)	<.01*
eGFR at the initiation of dialysis, median (IQR)	8.3 (6.4-10.6)	7.6 (5.8-10.2)	8.8 (7.0-11.1)	<.01*
Started dialysis as an inpatient, n (%)	461 (42)	208 (40)	253 (45)	.10
Started dialysis in the ICU, n (%)	57 (5)	26 (5)	31 (5)	.71
Any predialysis care, n (%)	1000 (92)	472 (90)	528 (93)	.07
Predialysis care ≥ 4 months, n (%)	842 (77)	396 (76)	446(79)	.23
Predialysis care ≥ 12 months, n (%)	678 (62)	305 (58)	373 (66)	.01*
Anatomical location of first fistula creation attempt, n (%):				<.01*
Radiocephalic	368 (34)	198 (38)	170 (30)	
Brachiocephalic/brachiobasilic	398 (36)	162 (31)	236 (42)	
Unknown/other	325 (30)	164 (31)	161 (28)	
First fistula attempt pre-dialysis, n (%)	508 (47)	223 (43)	285 (50)	.01*
Time from attempt to dialysis start, median (IQR)	145 (69-357)	138 (57-368)	157 (70-347)	.28
First fistula attempt post-dialysis, n (%)	583 (53)	301 (57)	282 (50)	.01*
Time from dialysis start to attempt, median (IQR)	99 (54-190)	96 (56-191)	103 (49-188)	

*Note.* IQR = interquartile range; BMI = body mass index; eGFR = estimated glomerular filtration rate; ICU = intensive care unit.

* Significant at *P* < .05.

**Table 2.** Catheter-Free Fistula Use and Access-Related Procedures, by Age Group.

All patients who attempted fistula creation	Age < 65 n = 524	Age ≥ 65 n = 567	*P* value
Median follow-up time after dialysis initiation (y)^a^, median (IQR)	1.8 (0.9-3.4)	2.0 (0.9-3.3)	.83
Fistula creation prior to start of dialysis, n (%)	223 (43)	285 (50)	.01*
Achieved catheter-free use of first fistula, n (%)	298 (57)	321 (57)	.93
Adjusted^b^ OR	Reference	1.01	.93
Achieved catheter-free use of any fistula, n (%)	345 (66)	354 (62)	.24
Adjusted^b^ OR	Reference	0.87	.36
Percentage of follow-up time with catheter-free use of first fistula, median (IQR)	19 (0-86)	21 (0-89)	.85
Adjusted^b^ OR	Reference	1.01	.91
Percentage of follow-up time with catheter-free use of any fistula, median (IQR)	40 (0-90)	36 (0-92)	.76
Adjusted^b^ OR	Reference	0.96	.73
Total rate of access procedures, per person-year (95% CI)	2.3 (2.2-2.4)	2.2 (2.1-2.3)	.04*
Adjusted^b^ IRR	Reference	0.95	.32
Catheter-related	1.1 (1.0-1.2)	0.93 (0.89-0.98)	<.01*
Adjusted^b^ OR	Reference	0.88	.13
Fistula-related (except fistula creations)	0.77 (0.72-0.81)	0.85 (0.80-0.89)	.01*
Adjusted^b^ OR	Reference	1.12	.14
Fistula creations	0.45 (0.42-0.49)	0.42 (0.39-0.46)	.22
Adjusted^b^ OR	Reference	0.93	.21
Restricted to patients who achieved catheter-free use	Age < 65 n = 298	Age ≥ 65 n = 321	
Percentage of follow-up time with catheter-free use of first fistula, median (IQR)	80 (46-100)	85 (48-100)	.85
Adjusted^b^ OR	Reference	1.04	.77
	n = 345	n = 354	
Percentage of follow-up time with catheter-free use of any fistula, median (IQR)	77 (44-100)	84 (47-100)	.18
Adjusted^b^ OR	Reference	1.07	.58

*Note.* IQR = interquartile range; OR = odds ratio; CI = confidence interval; IRR = incidence rate ratio.

a Follow-up time defined as the time between starting hemodialysis and any of the following: of death, kidney transplantation, lost to follow-up, transfer out of the program, start of peritoneal dialysis, recovery of kidney, or the end of the study period.

b All adjusted values are adjusted for sex, body mass index, diabetes mellitus, coronary artery disease, congestive heart failure, peripheral vascular disease, cerebrovascular disease, and whether fistula was created pre- or post-dialysis initiation.

* Significant at *P* < .05.

### The Impact of Age on the Likelihood of Catheter-Free Fistula Use

Only 57% of first fistula attempts resulted in catheter-free fistula use in both age groups, and there was no difference in the adjusted odds of achieving catheter-free use (OR_≥65vs<65_1.01; *P* value = .93) (Table 2). The median number of days of catheter-free use with the first fistula was similar (<65 years: 58 days vs ≥65 years: 52 days; *P* value = .82), as was the median percentage of follow-up time spent catheter-free (<65 years: 19% vs ≥65 years: 21%; *P* value = .85). If multiple fistula attempts were considered, the proportion of follow-up time spent catheter-free increased to 40% for those <65 years of age and 36% for those ≥65 years of age (adjusted OR_≥65vs<65_: 0.96; *P* value = .73).

Our modeled results were unchanged when we restricted the cohort to those with at least 3 years of follow-up, although the proportion of time spent catheter-free increased with increasing follow-up. In this selected cohort, when all fistula creations were considered, 73% of follow-up was spent catheter-free in patients <65 years of age compared with 75% in those ≥65 years. When age was categorized as <65, 65-75, and 75+, there was no significant difference in results by group (Table 3).

**Table 3.** Catheter-Free Fistula Use and Access-Related Procedures When Age Is Categorized Into 3 Groups (<65, 65-75, 75+ Years of Age).

	Age <65 (n = 524)	Age 65-75 (n = 297)	Age 75+ (n = 270)	*P* value
Achieved catheter-free use of first fistula, n (%)	298 (57)	170 (57)	151 (56)	.95
Adjusted^a^ OR	Reference	1.10	0.91	.56, .59
Achieved catheter-free use of any fistula, n (%)	345 (66)	185 (62)	169 (63)	.50
Adjusted^a^ OR	Reference	0.91	0.83	.57, .31
Percentage of follow-up time with catheter-free use of first fistula, median (IQR)	19 (0-86)	23 (0-87)	20 (0-94)	.87
Adjusted^a^ OR	Reference	1.06	0.96	.70, .80
Percentage of follow-up time with catheter-free use of any fistula, median (IQR)	40 (0-90)	34 (0-87)	41 (0-98)	.59
Adjusted^a^ OR	Reference	0.96	0.97	.74, .82
Total rate of access procedures, per person-year (95% CI)	2.3 (2.2-2.4)	2.4 (2.3-2.5)	2.0 (1.9-2.1)	.02*
Adjusted^a^ IRR	Reference	1.0	0.88	.96, .06

*Note.* IQR = interquartile range; OR = odds ratio; CI = confidence interval; IRR = incidence rate ratio.

a All adjusted values are adjusted for sex, body mass index, diabetes mellitus, coronary artery disease, congestive heart failure, peripheral vascular disease, cerebrovascular disease, and whether fistula was created pre- or post-dialysis initiation.

* Significant at *P* < .05.

**Figure 2.**
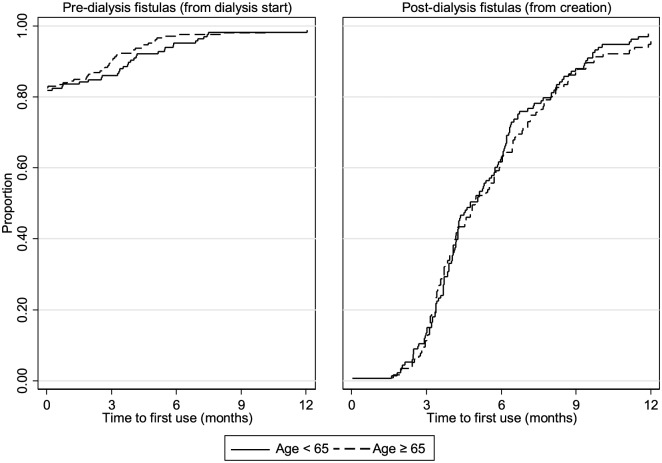
Time to catheter-free use of first fistula (months), among those who achieved it. *Note.* Analysis is restricted to patients who eventually achieved catheter-free use. Left panel is for predialysis fistula attempts with follow-up starting from dialysis initiation. Right panel is for postdialysis attempts with follow-up starting from creation date. Solid line represents individuals under age 65 years; dotted line represents individuals 65 years of age, and older.

Restricting the analysis to only those patients who successfully achieved catheter-free fistula use (n = 619), the median time to catheter-free use for predialysis fistulas was 0 days for both groups (indicating over 75% of successful predialysis fistula attempts were used for dialysis initiation) and 263 days for postdialysis fistula creations (<65 years: 241 days vs ≥65 years: 272 days; *P* value = .55)(see Figure 2). The median number of days of catheter-free use with the first fistula was similar (<65 years: 449 days vs ≥65 years: 525 days; *P* value: .42), as was the median percentage of follow-up time spent catheter-free (<65 years: 80% vs ≥65 years: 85%; *P* value: .85). If multiple fistula attempts were considered, the proportion of follow-up time spent catheter-free was similar, 77% for those <65 years of age and 84% for those ≥65 years of age (adjusted OR_≥65vs<65_: 1.07; *P* value = .58).

**Figure 3.**
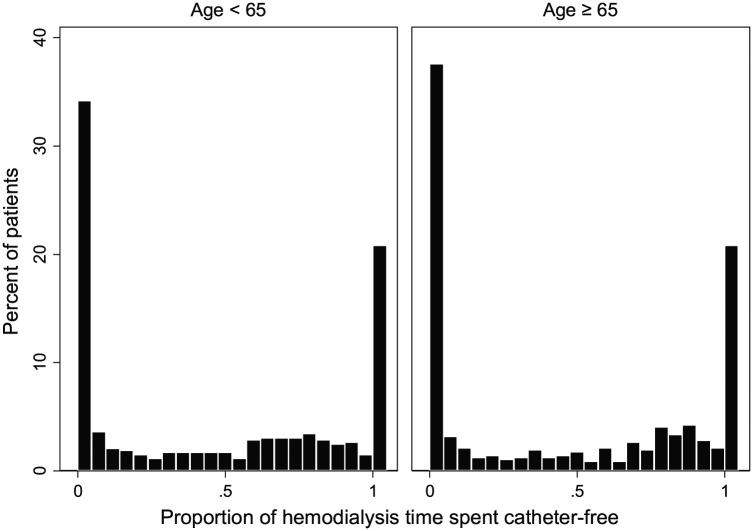
The proportion of time spent catheter-free in patients attempting a fistula creation. *Note.* The distribution of the proportion of time spent catheter-free was bi-modal in appearance. In both age groups, 21% used their fistula catheter-free for the entire duration of follow-up. However, a total of 34% in those <65 never used their fistula catheter-free compared with 38% of those ≥65 years.

When all patients who attempted a fistula were considered, a detailed look at individual patient experience showed that 21% attempted a fistula and used it for their entire dialysis career, 34% to 38% never used their fistula despite multiple attempts, and the remaining patients used a combination of catheters and fistulas (Figure 3).

### The Impact of Age on Access-Related Procedure Rates

The crude rates of access-related procedures were similar for both age groups (<65: 2.3 procedures per person-year vs ≥65: 2.2 procedures, per person-year, *P* value = .04), and adjusted rate ratios were not significantly different according to age group (overall, or by type) (Table 2).

## Discussion

In our study, 57% of patients who attempted fistula creation went on to catheter-free use of their fistulas. Older patients who underwent a fistula attempt were just as likely as younger patients to achieve catheter-free fistula use and achieved it at the same pace, after accounting for other patient characteristics. Furthermore, the duration of fistula use and the proportion of the dialysis career spent catheter-free were similar, regardless of age, as were adjusted procedure rates. Only 19% to 21% of the time spent on hemodialysis was catheter-free following a first fistula creation. This increased to 36% to 40% if multiple attempts at fistula creation were allowed and to 73% to 75% if analyses were restricted to a selected cohort that had at least 3 years of follow-up.

Studies looking at the impact of age on the likelihood of successful fistula maturation and use have yielded conflicting findings. Some have shown that older age adversely impacts fistula maturation and is accompanied by a higher rate of primary and secondary failure.^13,16^ For example, the REDUCE-FTM I study found patients older than 65 were less likely to mature their fistulas compared with their younger counterparts.^17^ A meta-analysis by Lazarides et al reported higher rates of primary failure at 12 and 24 months among elderly patients.^18^ However, other studies have suggested that age does not have an important impact on fistula outcomes.^19-22^ In a retrospective study of 658 patients referred for fistula creation, Weale et al^22^ assessed the impact of age on functional outcomes and found no difference in primary or secondary patency with radiocephalic and brachiocephalic fistulas in patients less than 65 years of age, 65 to 69 years of age, and those 80 years of age and older. Inconsistency in prior studies may relate to sample size considerations, a preponderance of single-center studies, differences in definitions, and the lack of adequate risk adjustment for other important predictors of fistula outcomes.^23,24^ Indeed, many risk factors for fistula failure are more common in older patients. We found that age was not an independent predictor of catheter-free fistula use. However, a smaller percentage of patients over age 65 underwent fistula creation, so the results are applicable to that selected population. It could be argued that a higher percentage of the population undergoes a fistula attempt in environments where fistulas are heavily promoted and that if more marginal candidates are included, the impact of age may be more pronounced. However, that would suggest that comorbidities and factors that negatively influence the likelihood of fistula maturation, rather than age itself, would be responsible.

Catheter-free fistula use was selected as our primary outcome of interest. Prior studies have focused on standard definitions of usability, and primary and secondary patency of fistulas.^13-21^ We opted for a more pragmatic definition of successful fistula creation. The expressed intent of fistula creation is to spare patients of the complications associated with indwelling catheters. As a consequence, successful fistula creation should lead to independent use of the fistula for the provision of dialysis. Furthermore, definitions of fistula maturation or patency based on numbers of runs where it is usable, with two-needles, at a predefined blood flow, over a specified period of time are cumbersome and difficult to apply for the purposes of ongoing quality improvement in dialysis programs. They may be very relevant in programs that are struggling with unsuccessful fistula placement to better understand where they need to intervene to improve fistula outcomes, but independent fistula use likely should be the ultimate measure of success if the intent is to spare patients of the risks associated with catheters.

The granularity of our data provided the opportunity to examine the outcomes of fistula attempts in detail. Despite an attempt at a first fistula creation, only 19% to 21% of patients’ dialysis careers are spent catheter-free. If patients have multiple attempts at a functioning fistula, the percentage of time spent catheter-free increases modestly to 36% to 40%. Furthermore, a more detailed look at individual patient experience is illuminating: 21% percent will attempt a fistula and use it for their entire dialysis career; 34% to 38% will never use their fistula despite multiple attempts; and the remaining patients will use a combination of catheters and fistulas. Thus, if the expressed purpose of attempting fistulas is to avoid catheter use, we are not successful in the vast majority of patients. One potential explanation for the disappointing numbers observed is the fact that half of the fistula attempts occurred after the start of dialysis. By the time a referral for fistula creation occurs, the operation is performed, and the fistula matures to a point that is usable, a significant period of time with a catheter has been accrued. Even with approximately 2 years of follow-up time, this may skew our results. Indeed, when we restricted our cohort to those with at least 3 years of follow-up, the proportion of time spent catheter-free increased to 78% and 74% in the <65 and ≥65 age groups, respectively. This may reflect the need for longer follow-up, or the fact that healthier individuals with healthier vessels are more likely to survive longer and contribute to the prevalent fistula population. These findings highlight the need to improve patient selection for fistula creation. If we could reliably identify those who were destined for success or failure, we would likely improve the patient experience, the efficiency of vascular access care, and possibly, patient outcomes. Unfortunately, a robust, generalizable method for patient selection has eluded us and current guidelines suggest fistulas for all patients. Future work will attempt to tease out the relative success of predialysis fistula creation versus creation of fistulas after the start of dialysis.

Our study has several strengths. It is a multicenter study and data were granular, prospectively collected, and coded using a common framework and definitions. The oversight over data collection was rigorous and data were double-reviewed by experts with queries sent to users to be corrected prior to analysis. The validity of data used in many prior studies is unknown. Finally, the sample size is large relative to other studies that employed primary clinical data collection.

Our study also has limitations. The median duration of follow-up in our cohort was 2 years. It may be that the benefits of fistula creation are underestimated due to the relatively short duration of follow-up. In addition, the fact that a smaller percentage of patients undergo fistula creation in the participating centers compared with other jurisdictions may influence the observed results. However, this is representative of Canadian practice and if centers are more selective when referring patients for a fistula attempt, our results likely represent a more positive view of fistula outcomes than are achieved in places where fistulas are more aggressively pursued. The observational design of our study means that we cannot make definitive conclusions about the utility of fistula creation in patients of any age group. A randomized trial is ultimately required to determine whether fistulas lead to better outcomes and to characterize the magnitude of that benefit, if present. A pilot randomized trial is currently underway to determine the feasibility of a larger trial comparing the outcomes of fistula creation to continued use of catheters in incident hemodialysis patients over the age of 65 in Canada (clinicaltrials.gov NCT02675569).^25^ We did not report the outcomes of patients treated with arteriovenous grafts. While there is increasing interest in the potential role of grafts in elderly patients, particularly in the United States, there are very few patients who undergo graft creation in a Canadian setting and our results may not be generalizable beyond current Canadian practice. Information about artery and vein size was not available. While we chose to classify patients according to age group for the reasons articulated, other approaches to determining biological age and suitability for fistula attempt may be more suitable and could be explored. Finally, we did not capture data about the patient perspective and the impact of fistula creation on patient-centered outcomes, which is an important consideration.

The policy implications of our findings are important. First, we have shown that age does not appear to be an important predictor of the success of fistula creation and probably should not be used alone to inform decision making about vascular access choice. Second, our results highlight that we do a relatively poor job at identifying patients who are a good candidate for fistula creation and are likely to experience good outcomes. There is clearly a subset of patients who experience a very uncomplicated course, but our ability to identify them a priori is currently poor. This is compounded by the fact that guidelines and quality improvement initiatives incent providers to attempt fistulas in all patients, rather than to be selective. Third, the fact that patients who attempt fistulas are still exposed to catheters for much of their dialysis careers speaks to the inefficiency of the current approach to patient selection. If the intent of fistula creation is to avoid exposure to catheters, our data would suggest that the current approach is not adequate.

In conclusion, age does not appear to be an important predictor of the success of fistula creation in hemodialysis patients. Only a small proportion of the time spent on hemodialysis was catheter-free in those who attempted fistula creations. Randomized comparisons are needed to establish the superiority of fistulas and better delineate the risks and benefits of various access strategies. Further work is needed to identify the subsets of patients who are likely to benefit from fistula creation to better inform patient selection.
